# Substrate specificity and proposed structure of the proofreading complex of T7 DNA polymerase

**DOI:** 10.1016/j.jbc.2022.101627

**Published:** 2022-01-22

**Authors:** Tyler L. Dangerfield, Serdal Kirmizialtin, Kenneth A. Johnson

**Affiliations:** 1 Department of Molecular Biosciences, Institute for Cellular and Molecular Biology, University of Texas, Austin, Texas, USA; 2 Chemistry Program, Science Division, New York University Abu Dhabi, Abu Dhabi, United Arab Emirates

**Keywords:** DNA polymerase, phosphorothioate, enzyme kinetics, proofreading, homology modeling, exonuclease proofreading structure, MD, molecular dynamics, TEAB, triethylammonium bicarbonate

## Abstract

Faithful replication of genomic DNA by high-fidelity DNA polymerases is crucial for the survival of most living organisms. While high-fidelity DNA polymerases favor canonical base pairs over mismatches by a factor of ∼1 × 10^5^, fidelity is further enhanced several orders of magnitude by a 3′–5′ proofreading exonuclease that selectively removes mispaired bases in the primer strand. Despite the importance of proofreading to maintaining genome stability, it remains much less studied than the fidelity mechanisms employed at the polymerase active site. Here we characterize the substrate specificity for the proofreading exonuclease of a high-fidelity DNA polymerase by investigating the proofreading kinetics on various DNA substrates. The contribution of the exonuclease to net fidelity is a function of the kinetic partitioning between extension and excision. We show that while proofreading of a terminal mismatch is efficient, proofreading a mismatch buried by one or two correct bases is even more efficient. Because the polymerase stalls after incorporation of a mismatch and after incorporation of one or two correct bases on top of a mismatch, the net contribution of the exonuclease is a function of multiple opportunities to correct mistakes. We also characterize the exonuclease stereospecificity using phosphorothioate-modified DNA, provide a homology model for the DNA primer strand in the exonuclease active site, and propose a dynamic structural model for the transfer of DNA from the polymerase to the exonuclease active site based on MD simulations.

DNA polymerases have evolved to efficiently copy genomes with extremely high fidelity to fulfill their critical role in maintaining genome stability. A large contribution to replication fidelity comes from a finely tuned polymerase active site that makes errors in only one out of 10^5^ base pairs while incorporating nucleotides at rates exceeding 300 per second (1, 2). High-fidelity polymerases also contain a 3′–5′ proofreading exonuclease that further increases replication fidelity by removing mismatches after they are incorporated. The contribution of this activity to fidelity varies depending on the enzyme (3), ranging from a factor of ∼10 to more than 10,000 (4, 5, 6). Early studies suggested that the substrate for the exonuclease reaction is at least partially single-stranded, rather than duplex DNA, based on the temperature dependence of the exonuclease reaction (7), but temperature dependence alone cannot distinguish alternative models because of expected differences in the thermodynamics of DNA binding at the pol (polymerase) and exo (exonuclease) sites. Before structures were available, early work was complicated by the low sequence identity of different exonucleases as sequence alignment of exonuclease domains from different enzymes usually gives less than 15% sequence identity (8, 9). Subsequent crystal structures and kinetic studies on T4 DNA polymerase and Klenow Fragment polymerase identified conserved active site carboxylates that coordinate two metal ions at the exo site, suggesting a general two-metal ion mechanism (9, 10). Studies on phosphorothioate DNA supported this mechanism and afforded insights into the stereochemistry of the reaction (11, 12, 13). Phosphorothioate-modified DNAs have been subsequently employed in biotechnology to increase the stability of nucleic acids *in vivo* (14).

The DNA polymerase from bacteriophage T7 has been an important model system for understanding high-fidelity DNA replication due to its simple structure consisting of only two polypeptides (phage T7 gene product 5 and *Escherichia coli* thioredoxin) and its fast, accurate, and processive DNA replication. We previously determined the complete kinetic pathway of correct nucleotide incorporation and misincorporation by T7 DNA polymerase using a variant with a fluorescent artificial amino acid (15, 16). This polymerase also features a 3′–5′ proofreading exonuclease active site within the N-terminal region of gene product 5, approximately 35 Å from the polymerase active site. Prior studies provided an initial kinetic model for exonuclease proofreading by this enzyme (4), showing that: 1) ssDNA binds directly to the exonuclease active site and is rapidly hydrolyzed at a rate of at least 1000 s^−1^; 2) a fraction of dsDNA substrate binds directly to the exonuclease active site and is rapidly hydrolyzed, while the remainder binds at the polymerase active site and then transfers to the exo site, which limits the rate of mismatch hydrolysis (Scheme in Fig. 1). The fraction that binds to each site and the rate of transfer between the two active sites are dependent on the extent of fraying of the primer terminus of the DNA substrate. Although it was shown that mismatches can be slowly extended by correct bases (*k*_*cat*_: 0.025 s^−1^ k_m_: 87 μM (17)) and are rarely extended by a mismatched nucleotide, important outstanding questions remain. In particular, the rate of extension relative to excision of these buried mismatches remains to be measured to assess the net contribution of the exonuclease to replication fidelity. Moreover, there is no structural model for the exonuclease–DNA complex of T7 DNA polymerase.

**Figure 1:**
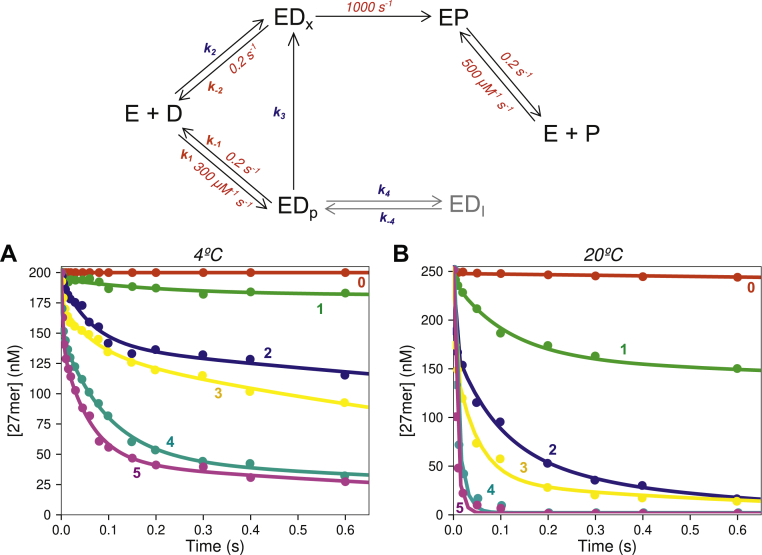
**Effect of temperature and number of terminal mismatches on excision kinetics.** Scheme: Kinetic model for exonuclease proofreading by the DNA polymerase. Rate constants with numeric values given in *red* were locked during data fitting at values defined by lower limits derived in preliminary data fitting as described in the main text. The remaining rate constants were variables during the final data fitting. Reaction details: A solution of 1 μM T7 DNA polymerase, 20 μM thioredoxin, 0.1 mg/ml BSA, and 12.5 mM Mg^2+^ was mixed with FAM-DNA (200 nM for 4 °C, 250 nM for 20 °C) to start the reaction in the quench flow instrument. *A*, concentration of starting DNA *versus* time for substrates with varying numbers of terminal mismatches at 4 °C. *B*, concentration of starting DNA *versus* time for substrates with varying numbers of mismatches at 20 °C. In both figures, the *colored numbers* represent the number of terminal mismatches in the substrates. The *solid lines* through the data are the best fits obtained using KinTek Explorer based on the model at the *top* of the figure. The relative amplitudes of the fast and slow phases define the kinetic partitioning in DNA binding to the exo or pol sites, respectively.

In this paper, we present our kinetic characterization of the substrate specificity of the proofreading exonuclease of T7 DNA polymerase. We address the temperature dependence of the proofreading reaction, measure the kinetics of extension *versus* excision of buried mismatches, and propose a structural model for the exonuclease active site based on homology modeling refined by MD simulation. Our data demonstrate that the fidelity of this enzyme may be much higher than that previously estimated and shows key active site differences when compared with other DNA polymerases with proofreading exonuclease domains.

## Results

### Temperature dependence of the exonuclease reaction on DNA with varying terminal mismatches

We performed our previous kinetic studies on the correct nucleotide incorporation for T7 DNA polymerase at 4 °C in order to better resolve the fast rate of the conformational change step, so we initially performed experiments to characterize the exonuclease activity at 4 °C to be consistent with the previous results (1). Our previous study and others (18, 19) showed that while the rates of polymerization are somewhat temperature-dependent, the specificity at the polymerase active site, defined by the relative *k*_*cat*_*/K*_*m*_ values for correct *versus* mismatched nucleotides, did not change significantly as a function of temperature (15) over physiological temperature ranges (4–20 °C studied). Early studies on exonuclease proofreading for Klenow Fragment (7) showed a strong temperature dependence of the reaction, which was attributed to the effect of temperature on the melting of mispaired DNA bases, but could be a function of other structural changes involved in transferring DNA from the polymerase to the exo sites. Moreover, the effect of temperature on the proofreading reaction for T7 DNA polymerase remains to be studied. To begin our studies on the proofreading exonuclease activity for T7 DNA polymerase, we performed single turnover rapid quench experiments with synthetic DNA substrates containing 0 through 5 terminal mismatches (Table 1) at 4 and 20 °C (Fig. 1). A 5′-6-carboxyfluorescein (6-FAM) label on the primer strand was used to quantify the reaction products by PAGE or by capillary electrophoresis (20). A molar excess of T7 DNA polymerase was mixed with the DNA substrate of interest to start the reaction, and samples were quenched at various times by addition of ethylenediamine tetraacetic acid (EDTA) (0.3 M). The loss of the starting material (27 nt primer) for each substrate was fit by simulation using the model shown in Figure 1. Best fits derived by simulation for each substrate are shown as the solid lines in Figure 1*A*, and the resulting rate constants are given in Table 2.

**Table 1:** Oligonucleotides used in this study

Oligonucleotide name	Oligonucleotide sequence (5′→3′)	ε_260 nm_ (M^−1^ cm^−1^)
FAM-27	[FAM]-CCGTCGCAGCCGTCCAACCAACTCAAC	266,660
45	GGACGGCATTGGATCGATGTTGAGTTGGTTGGACGGCTGCGACGG	433,700
45–1 MM	GGACGGCATTGGATCGAT**C**TTGAGTTGGTTGGACGGCTGCGACGG	430,200
45–2 MM	GGACGGCATTGGATCGAT**CA**TGAGTTGGTTGGACGGCTGCGACGG	435,500
45–3 MM	GGACGGCATTGGATCGAT**CAA**GAGTTGGTTGGACGGCTGCGACGG	439,400
45–4 MM	GGACGGCATTGGATCGAT**CAAC**AGTTGGTTGGACGGCTGCGACGG	435,700
45–5 MM	GGACGGCATTGGATCGAT**CAACT**GTTGGTTGGACGGCTGCGACGG	430,400
45-n-1-BMM	GGACGGCATTGGATCGATG**A**TGAGTTGGTTGGACGGCTGCGACGG	438,200
45-n-2-BMM	GGACGGCATTGGATCGATGT**A**GAGTTGGTTGGACGGCTGCGACGG	439,600
45-n-3-BMM	GGACGGCATTGGATCGATGTT**C**AGTTGGTTGGACGGCTGCGACGG	431,000
45-n-4-BMM	GGACGGCATTGGATCGATGTTG**T**GTTGGTTGGACGGCTGCGACGG	429,200
45-n-5-BMM	GGACGGCATTGGATCGATGTTGA**C**TTGGTTGGACGGCTGCGACGG	429,200
45-n-7-BMM	GGACGGCATTGGATCGATGTTGAGT**A**GGTTGGACGGCTGCGACGG	439,600
45-n-10-BMM	GGACGGCATTGGATCGATGTTGAGTTGG**A**TGGACGGCTGCGACGG	438,200
6mer PThio	CTCAA∗C	54,300

The ∗ symbol indicates a phosphorothioate linkage. The FAM-27 primer strand was used with each mismatch as defined by various template strands. Mismatches are shown in bold text.

**Table 2:** Kinetic parameters from fitting data with different numbers of mismatches at 4 °C

# Of mismatches	% Flux DNA to *ED*_*x*_*versus ED*_*p*_	% Flux *ED*_*p*_ to *ED*_*x*_*versus EI*	*k*_*3*_ (s^−1^)	*K*_*3*_	*k*_*2*_ (μM^−1^ s^−1^)	*k*_*4*_ (s^−1^)	*k*_*−4*_ (s^−1^)
0	<1	–	<0.01	0.0003	–	–	–
1	3	7	0.28 ± 0.10	0.033	9.8 ± 1.5	4.0 ± 1.9	<0.5
2	4	35	4.4 ± 0.7	0.042	12.7 ± 4.1	10.5 ± 2.6	1.0 ± 0.5
3	14	45	3.7 ± 0.7	0.157	47 ± 4	9.2 ± 3.0	2.6 ± 0.8
4	27	76	7.2 ± 0.3	0.360	108 ± 3	2.9 ± 0.5	0.72 ± 0.45
5	32	76	12.0 ± 0.7	0.463	139 ± 5	5.1 ± 0.8	1.13 ± 0.44

Rate and equilibrium constants are numbered according to the scheme in Figure 1 and were derived by global data fitting as described in the text. Rate constant *k*_*1*_ was locked at 300 μM^−1^ s^−1^ while *k*_*−1*_ and *k*_*−2*_ were locked at 0.2 s^−1^. *K*_*3*_ defines the equilibrium constant for transfer of the DNA from the pol to exo site. Percent flux was derived numerically from the partial derivatives in KinTek Explorer as described in Experimental procedures.

In the simple model in the scheme in Figure 1, adapted from earlier studies on exonuclease proofreading for T7 DNA polymerase (4), the DNA can bind either at the polymerase active site (with second order rate constant, *k*_*1*_) and then transfer to the exonuclease active site (*k*_*3*_), or the DNA can bind directly to the exonuclease active site (*k*_*2*_) where it is rapidly hydrolyzed at a rate of ∼1000 s^−1^ (4). This model explains the biphasic nature of the excision data, where the fast phase represents the fraction binding directly to the exonuclease active site where it is rapid excised, and the slower phase represents the fraction that binds at the polymerase active site and is more slowly transferred to the exonuclease active site. Initial attempts to fit the simple excision data to this model revealed that the individual rate constants for binding (*k*_*1*_, *k*_*2*_) and dissociation (*k*_*−1*_, *k*_*−2*_) at the exonuclease and polymerase active sites were not well constrained by the data. While the individual rate constants could take on a large number of values and still provide a reasonable fit, it was clear that the ratio of second order rate constants for binding at the two sites was well constrained by the data. Therefore, to extract the relevant information using a minimal model, we locked *k*_*1*_ at 300 μM^−1^s^−1^ as a reasonable estimate for diffusion-limited DNA binding (although all values from ∼50–600 μM^−1^s^−1^ gave a nearly identical fit), we locked *k*_*−1*_ and *k*_*−2*_ at 0.2 s^−1^ as reasonable minimal estimates for rates of DNA dissociation from each site (although values up to 50 s^−1^ depending on *k*_*1*_ and *k*_*2*_ gave reasonably good fits). We then allowed *k*_*2*_ to float in the fitting. This approach to global fitting allowed us to accurately extract the information content of the data; namely, the relative rates of binding to the pol and exo sites and the rate and equilibrium constants for transfer from the pol to the exo site. For each substrate, we then used the flux calculation feature of KinTek Explorer (see Experimental procedures) to determine the fraction of DNA that binds from solution to each site and the rate of transfer from the polymerase to the exonuclease active site. These are the principal parameters that were well defined by the data. The flux, rates of transfer between active sites, as well as the individual rate constants used in the flux calculation are given in Table 2. In addition to these parameters, another step was added that limits the fraction of hydrolyzed DNA in the first binding event since the curves did not trend toward complete reaction on the timescale of the experiments. We added a step where the ED_p_ state interconverts to an intermediate inhibited state, ED_I_, to account for the nonzero endpoint. The resulting rate constants for the partitioning of the ED_p_ state are given in Table 2. To simplify the interpretation, we performed flux calculations to determine the fraction of ED_p_ that partitions to ED_x_ *versus* ED_I_ (Table 2).

Although the biphasic nature of the curves is consistent with previous work, the activity we measured at 4 °C was significantly lower than that previously reported for this enzyme at 20 °C, as demonstrated by the data in Figure 1 and the quantification in Table 2. Even with five terminal mismatches, less than 35% of the DNA bound directly to the exonuclease active site from solution and a significant fraction (∼25%) partitioned into the inhibited state at 4 °C. We then repeated the above experiments at 20 °C to examine the effect of temperature on the proofreading reaction. DNA substrates with various numbers of mismatches were mixed with an excess of T7 DNA polymerase to start the reaction in the quench-flow instrument. Samples were processed as described above to give the results shown in Figure 1*B* with the fitted parameters shown in Table 3. We found much faster exonuclease activity at 20 °C, with rates comparable to those observed in earlier studies (4). When increasing the temperature from 4 °C to 20 °C (Tables 2 and 3), the rates of transfer from the polymerase to exonuclease active site increase by factors of 2 to 10, depending on the DNA substrate, while the fraction that bound directly to the exonuclease active site also increased by a factor ranging from two- to eightfold. Finally, a greater fraction partitioned from ED_p_ to ED_x_ relative to ED_I_ at 20 °C, accounting for the observation that the reaction proceeded more closely toward completion at the higher temperature.

**Table 3:** Kinetic parameters from fitting data with different numbers of mismatches at 20 °C

# Of mismatches	% Flux DNA to *ED*_*x*_*versus ED*_*p*_	% Flux *ED*_*p*_ to *ED*_*x*_*versus EI*	*k*_*3*_ (s^−1^)	*K*_*3*_	*k*_*2*_ (μM^−1^ s^−1^)	*k*_*4*_ (s^−1^)	*k*_*−4*_ (s^−1^)
0	<1	–	0.023 ± 0.006	0.0087	2.6 ± 1.6	–	–
1	6	38	2.9 ± 0.3	0.061	18.3 ± 3.5	5.42 ± 0.73	0.33 ± 0.05
2	33	78	7.7 ± 0.7	0.473	142 ± 7	2.22 ± 0.96	3.2 ± 1.2
3	37	82	16.6 ± 1.8	0.573	172 ± 11	3.9 ± 1.2	1.8 ± 0.7
4	50	100	69 ± 8	1.003	301 ± 25	–	–
5	59	100	102 ± 10	1.400	420 ± 28	–	–

Rate and equilibrium constants are numbered according to the scheme in Figure 1 and were derived by global data fitting as described in the text. Rate constant *k*_*1*_ was locked at 300 μM^−1^ s^−1^ while *k*_*−1*_ and *k*_*−2*_ were locked at 0.2 s^−1^. *K*_*3*_ defines the equilibrium constant for transfer of the DNA from the pol to exo site. Percent flux was derived numerically from the partial derivatives in KinTek Explorer as described in Experimental procedures.

Inclusion of the ED_I_ state is necessary to construct a minimal model that accurately describes the data, but its identity is unknown. Transfer of DNA to the exo site presumably depends on the fraction of frayed primer at the pol site, so the ED_I_ state could be due to a small fraction of nonfrayed DNA that is stabilized at the pol site, perhaps by a conformational change in the enzyme corresponding to the nucleotide-induced closed state (1). Previously we calculated the fidelity of wild-type T7 DNA polymerase at the polymerase active site at 4 °C (15) and found that it was comparable with the value measured at 20 °C (17), indicating that despite changing rate constants, the overall fidelity of the polymerase during incorporation was not significantly affected by temperature. This unexpected result is consistent with the broad physiological temperature range for bacteriophage T7. However, we observed a large effect of temperature on the proofreading activity, presumably reflecting the significant conformational changes in the enzyme and DNA melting required to transfer the DNA primer strand from the polymerase to the exonuclease active site approximately 35 Å away. Because of the strong temperature dependence on the proofreading reaction between 4 °C and 20 °C, the rest of the experiments in this paper were performed at 20 °C to more closely mimic standard physiological conditions.

### Proofreading kinetics on substrates with mismatches buried by correct bases

In the previous section, we showed that DNA substrates containing more terminal mismatches were excised faster than DNA substrates with fewer mismatches. This is not surprising as mismatched primer termini are typically frayed and are expected to more readily bind to the exonuclease active site (4). As the temperature increases, so does the degree of fraying of the mismatch and correspondingly more of the primer strand partitions into the exonuclease active site. However, the probability of creating such a substrate with multiple terminal mismatches *in vivo* is highly unlikely for this enzyme. The experiments in Figure 1 were designed to probe the underlying mechanisms rather than represent physiologically relevant states. A more likely scenario is that after misincorporation, the mismatch may either be removed or extended by the addition of the next correct nucleotide (17), potentially allowing further polymerization of more correct nucleotides and in turn, burying the mismatch so a mutation becomes incorporated into the genome.

**Figure 2:**
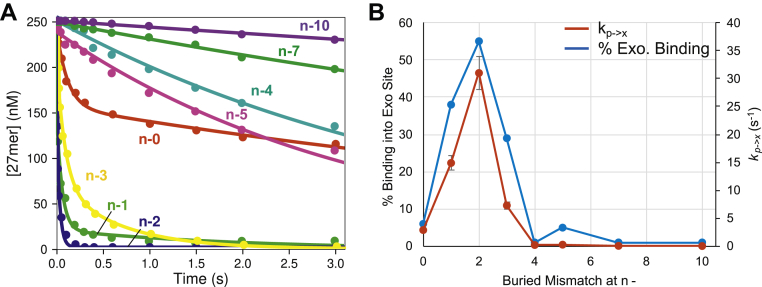
**Effect of buried mismatch position on excision kinetics.** Reaction conditions: A solution of 1 μM T7 DNA polymerase, 20 μM thioredoxin, 0.1 mg/ml BSA, and 12.5 mM Mg^2+^ was mixed with 250 nM FAM-DNA to start the reaction in the quench flow instrument at 20 °C. The reaction was stopped by mixing with 0.3 M ethylenediamine tetraacetic acid, and the products were resolved by capillary electrophoresis. *A*, concentration *versus* time for mismatches buried by zero (n-0) to 10 nucleotides (n-10). *Colored labels* indicate the position of the buried mismatch in the DNA substrate. *Colored lines* through the data are best fits by simulation in using the model in Figure 1. The n-0 mismatch is the same data as shown for the single 3′ mismatch substrate in Figure 1*B*. *B*, percentage of DNA binding into the exonuclease active site and rate of transfer between sites for various buried mismatches. The percentage bound into the exonuclease active site (*blue*) was derived from the amplitude of the fast phase in *A* and rates of transfer from polymerase to exonuclease active sites (*red*) were derived from the rate of the slow phase (*k*_*p->x*_ = *k*_*3*_ in Fig. 1). These values and flux calculations (Table 2) were calculated from parameters derived during data by simulation using KinTek Explorer with the model in Figure 1. Error bars for *k*_*p->x*_ are from the standard errors derived from fitting the data.

To assess whether the exonuclease was afforded multiple opportunities for proofreading after a single mismatch, we compared rates of excision of mismatches buried by different numbers of correct base pairs. Rapid quench experiments (at 20 °C) were performed as before by mixing DNA with an excess of T7 DNA polymerase to start the reaction. The data were analyzed as described in the previous section. Figure 2*A* shows the time course of excision for the various buried mismatches tested, with mismatches at various positions from n-1 to n-10 relative to the 3′ end of the primer, in addition to a 3′ terminal mismatch (n-0). Data were fit by simulation using the model in Figure 1, as described above, to derive the fraction of DNA that binds directly to the exonuclease active site *versus* the polymerase active site, the flux from ED_p_ to ED_x_ relative to ED_I_, and the rate of transfer from the polymerase to the exonuclease active site. Rate constants and flux parameters estimated from the fitting are given in Table 4. Surprisingly, mismatches buried at the n-1 through n-3 position were removed even more efficiently than the 3′-terminal mismatch (n-0). The fraction that bound to the exo site from solution relative to the pol site was 5 to 9 times greater than for the terminal mismatch, and the rate of transfer from ED_p_ to ED_x_ was 2 to 5 times greater than the substrate containing a 3′-terminal mismatch (n-0). Substrates with buried mismatches at the n-4 and n-5 positions, however, were removed slower than a terminal mismatch. Minimal exonuclease activity was observed for the n-7 and n-10 buried mismatch substrates, although these substrates were hydrolyzed at rates slightly faster than the substrate with 0 mismatches. These results suggest that the mechanisms governing how mutations are removed or stably incorporated into the genome by this high-fidelity DNA polymerase must be re-evaluated. Proofreading appears to remove a mismatch most efficiently after it has been buried by incorporation of correct base pairs. However, to fully evaluate the fate of a mismatch, we need to measure rates of extension and excision.

**Table 4:** Kinetic parameters from fitting data at different buried mismatch position at 20 °C

Buried mismatch position	% Flux DNA to *ED*_*x*_*versus ED*_*p*_	% Flux *ED*_*p*_ to *ED*_*x*_*versus EI*	*k*_*3*_ (s^−1^)	*K*_*3*_	*k*_*2*_ (μM^−1^ s^−1^)	*k*_*4*_ (s^−1^)	*k*_*−4*_ (s^−1^)
n-0 (3′ mismatch)	6	35	2.9 ± 0.3	0.061	18.3 ± 3.5	5.42 ± 0.73	0.33 ± 0.05
n-1	38	88	14.9 ± 1.3	0.597	179 ± 11	2.33 ± 0.58	0.62 ± 0.27
n-2	55	100	31 ± 3	1.19	357 ± 24	–	–
n-3	29	72	7.3 ± 0.6	0.393	118 ± 6	3.0 ± 0.7	2.3 ± 0.4
n-4	<1	–	0.22 ± 0.01	0.007	<2	–	–
n-5	5	–	0.29 ± 0.01	0.049	14.7 ± 3.1	–	–
n-7	<1	–	0.078 ± 0.003	0.007	<2	–	–
n-10	<1	–	0.028 ± 0.001	0.007	<2	–	–

Rate and equilibrium constants are numbered according to the scheme in Figure 1 and were derived by global data fitting as described in the text. Rate constant *k*_*1*_ was locked at 300 μM^−1^ s^−1^ while *k*_*−1*_ and *k*_*−2*_ were locked at 0.2 s^−1^. *K*_*3*_ defines the equilibrium constant for transfer of the DNA from the pol to exo site. Percent flux was derived numerically from the partial derivatives in KinTek Explorer as described in Experimental procedures.

### Buried mismatch extension for T7 DNA polymerase

It is known that DNA polymerases can extend terminal mismatches with correct bases, albeit at much lower efficiency than sequential correct nucleotide incorporation (17). In further studies (Fig. S1 and Table S1), we found that buried mismatched were also extended inefficiently relative to correct nucleotides. The observed *k*_*cat*_/*K*_*m*_ values were similar to what was previously measured for 3′-terminal mismatches, although individual *k*_*cat*_ and *K*_*m*_ values varied widely. For this high-fidelity enzyme, the rate constant for correct incorporation on top of a mismatch is approximately 12,000 times lower than the rate constant for extending a correct base pair. It generally has been assumed that after mismatch extension with a correct base, the polymerase can continue rapid replication by adding more correct bases to bury the mismatch, but rates of buried mismatch extension have not been measured. If buried mismatch extension is fast relative to excision, this paradigm holds, and a mutation would be added to the genome after extending the mismatch with a single correct base. However, if the rate of buried mismatch extension is greatly hindered, then the fast rate of excision on these substrates could provide a mechanism to further increase the replication fidelity of this enzyme by affording multiple opportunities for proofreading.

**Figure 3:**
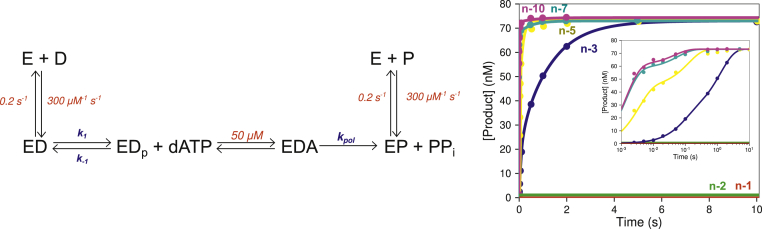
**Buried mismatch extension kinetics.** Scheme: ED represents an initial collision complex for DNA binding prior to docking into the polymerase site (ED_p_). Rate constants shown in *red* were locked at the values shown during the fitting as described in the text. The remaining rate constants were allowed to float during the fitting. Concentration *versus* time plot: Reaction conditions: A solution of 200 nM exo^−^ T7 DNA polymerase, 4 μM thioredoxin, 0.1 mg/ml BSA, and 75 nM FAM-DNA was mixed with 500 μM dATP and 12.5 mM Mg^2+^ to start the reaction in the quench flow instrument. The reaction was stopped at various times by mixing with 0.3 M ethylenediamine tetraacetic acid, and the products were resolved by capillary electrophoresis. The concentration of extended DNA product *versus* time is shown for buried mismatches at the n-1 through n-10 position. Rates of extension are given in Table 5. Inset: product *versus* time data, shown on a logarithmic timescale.

To investigate mismatch extension with correct bases, we performed single turnover experiments to directly measure rates of extension. To measure rates of extension without complications caused by the exonuclease reaction, we use an exo^−^ variant (D5A/E7A) (21), which exhibited polymerization kinetics comparable with wild-type enzyme (4). Experiments using this variant therefore allow analysis of reactions at the polymerase active site without complications from the exonuclease reaction. A solution of the exo^−^ T7 DNA polymerase variant was preincubated with DNA substrates containing buried mismatches at various positions and was then mixed with 500 μM dATP (the next correct base) to start the extension reaction (Fig. 3). Reaction products were then resolved by either capillary electrophoresis or PAGE, and the data were fit by simulation using the model given in the scheme shown in Figure 3. This model mimics the experiment by allowing equilibration of the DNA between the exonuclease and polymerase active sites before adding nucleotide to provide an estimate of the fraction of DNA in the state primed for polymerization and the observed rate of incorporation catalyzed by the fraction of enzyme in that state. Thus, at a minimum we can approximate the rates of extension at the pol site while acknowledging that the partitioning to the exo site may be affected by the exo^−^ double mutation.

For the n-1 and n-2 buried mismatch substrates, no extension was observed even after 180 s and up to 1 mM dATP, putting an upper limit of *k*_*cat*_/*K*_*m*_ < 0.1 M^−1^ s^−1^ for extension of these buried mismatch substrates. Data for extension of the n-3 buried mismatch data are biphasic, with approximately 1/3 of the enzyme–DNA complex primed for polymerization upon nucleotide addition (Table 5) with a rate of extension of 13.2 s^−1^ at 500 μM ATP. For the n-5 buried mismatch, approximately 60% of the DNA was extended at a rate greater than 400 s^−1^, consistent with rates measured on a DNA substrate without any mismatches (21). The n-7 and n-10 buried mismatch substrates were also extended at greater than 400 s^−1^, with approximately 93% and 98% of the DNA at the pol site in a state primed for nucleotide incorporation, respectively. Thus, the enzyme fails to detect a mismatch only after it has been buried by 5 to 7 nucleotides.

**Table 5:** Kinetic parameters from fitting buried mismatch extension data^a^

Buried mismatch position	*k*_*pol*_ (s^−1^)	Fraction primed for polymerization	*k*_*1*_ (s^−1^)	*k*_*−1*_ (s^−1^)
n-1	<0.001	–	–	–
n-2	<0.001	–	–	–
n-3	13.2 ± 9.6	36	0.82 ± 0.25	1.46 ± 1.38
n-5	400 (locked)	61	8.1 ± 5.2	5.2 ± 4.1
n-7	400 (locked)	93	2 (ND)	0.15 (ND)
n-10	400 (locked)	98	1.5 (ND)	0.04 (ND)

a For parameters shown as –, the step was not included in the model. For parameters followed by (ND), standard errors are not reported because the individual rate constants were not well constrained by the data, although the ratio of values was defined by data fitting.

The n-1 through n-3 buried mismatch substrates showed high proofreading efficiency but almost no extension. These observations lead to a model where the enzyme stalls after incorporation of a mismatch, affording multiple opportunities for proofreading. Even in the rare case that the buried mismatch is extended, it is much more likely to be excised by the proofreading exonuclease than to be extended; therefore, virtually all mismatches would be removed. This model suggests that the contribution of the exonuclease domain to fidelity may be much higher than previously estimated (4), at least on undamaged DNA and without other mutagenic factors.

To quantitatively assess the implications of our findings, we performed a simulation in KinTek Explorer with the parameters derived above to determine the fate of a substrate containing a single terminal mismatch. The simulation showed that approximately 99.5% of the single mismatch was excised by the proofreading exonuclease domain while the remaining 0.5% was extended with the correct base to form the DNA substrate with a buried mismatch, consistent with previous estimates (4). Since we only obtained an upper limit on the rate of extension of the buried mismatch, this upper limit was used in the calculation for buried mismatch partitioning. The simulation showed that less than 1% of the original 0.5% of the mismatch that got extended was further extended to the n-2 buried mismatch substrate, suggesting less than 0.005% of the original mismatch would be buried and incorporated into the genome. Taken together, these numbers suggest that efficient buried mismatch excision *versus* extension gives an overall 10^4^-fold contribution of the proofreading exonuclease to DNA replication fidelity. At this rate, mutations would almost never be incorporated into the T7 genome during replication. However, it is important to note that we have only investigated a single sequence context in undamaged DNA in this study, and it is likely that different sequence contexts, damaged DNA, or the presence of other replication complex proteins could lower the actual fidelity of DNA replication *in vivo*. Nonetheless, the innate speed and selectivity of the exonuclease domain of T7 DNA pol reveal a highly efficient system for removal of mismatches.

### Homology model for T7 DNA polymerase exonuclease complex

The activity of exonuclease domains from other DNA polymerases (Klenow Fragment (10, 11, 22, 23), RB69 polymerase (24, 25, 26, 27), T4 polymerase (3, 26, 28, 29, 30, 31, 32, 33, 34, 35, 36, 37), polymerase γ (38), and others (39, 40, 41)) has been extensively characterized. Structural data for proofreading complexes for most DNA polymerases (including T7 DNA polymerase) are lacking unlike the large number of structures with DNA in the polymerase active site. The large distance between the exonuclease and polymerase active sites (∼35 Å) for T7 DNA polymerase poses important unresolved questions regarding the mechanism of transfer between the two sites. We created a structural model for a proofreading complex of T7 DNA polymerase with the primer strand of a dsDNA substrate in the exonuclease active site based on homology to the crystal structure of a similar complex for Klenow Fragment (42). While Klenow Fragment is also an A family polymerase like T7 polymerase with high structural homology (but relatively low sequence identity), it is a low-fidelity repair enzyme where the exonuclease domain only contributes a factor of ∼10 to replication fidelity (43). However, it is the closest exonuclease structure to T7 DNA polymerase available. After building a homology model, we refined the structure with all atom molecular dynamics (MD) simulations with the program GROMACS (44).

**Figure 4:**
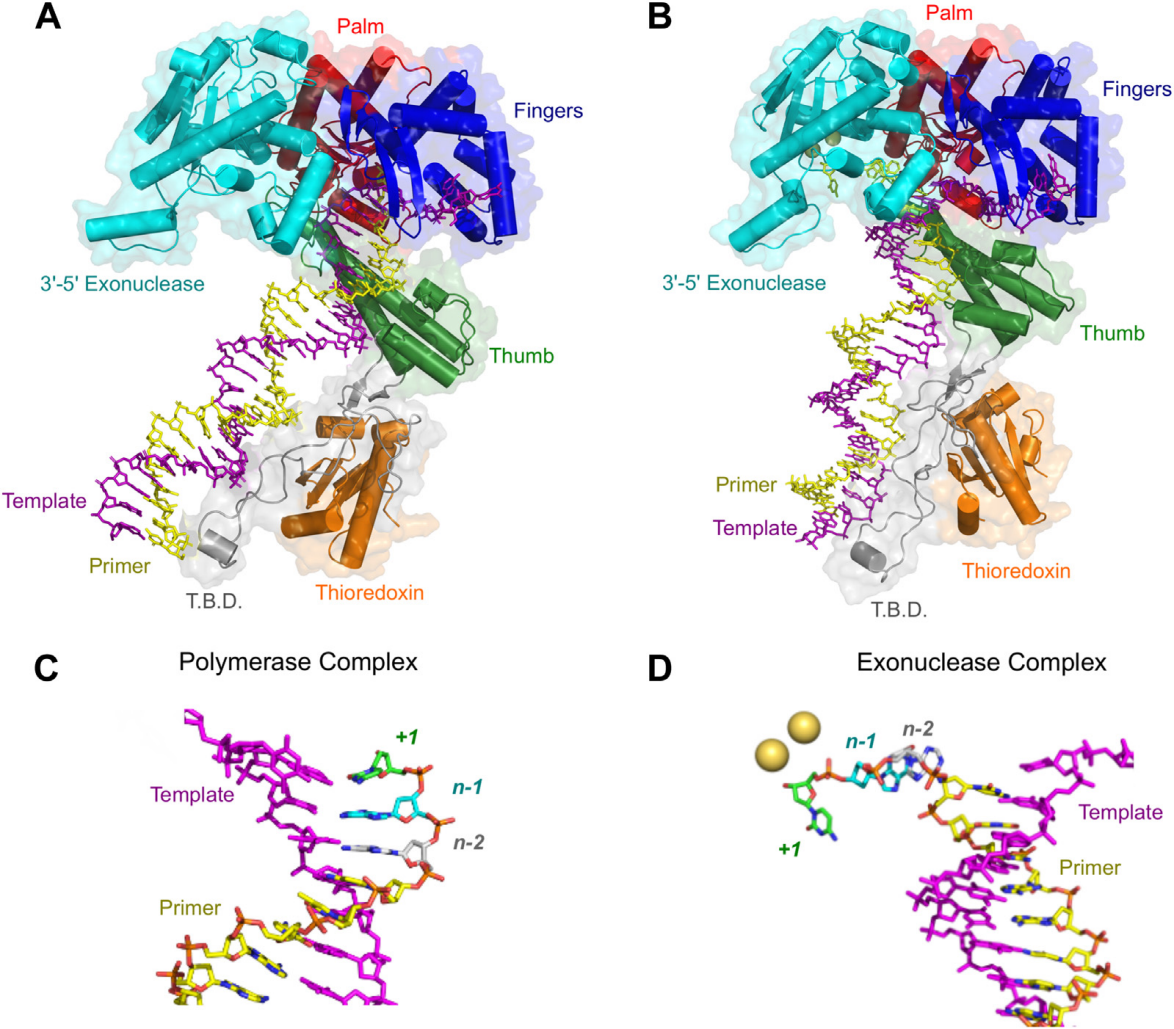
**Structural model for exonuclease site of T7 DNA polymerase.** Structures in this figure are from the all-atom MD simulations as described in the main text. *A*, overall model for “Polymerase Complex” from MD simulations. This complex corresponds to the structure with the primer in the polymerase active site between the fingers, thumb, and palm domains. Domain labels are in the same color of the respective domain. *B*, overall model for “Exonuclease Complex” from MD simulations. This complex corresponds to the structure with the primer strand in the exonuclease active site, which was determined based on homology to the Klenow Fragment proofreading complex structure (42). Domain labels are in the same color of the respective domain. *C*, DNA from “Polymerase Complex”. The template strand is shown in *magenta*, and the primer strand is colored in *yellow*. The three terminal bases of the primer strand from 3′-5′ are colored *green*, *cyan*, and *gray*, respectively. *D*, DNA from “Exonuclease Complex”. The template strand is shown in *magenta*, and primer strand is colored in *yellow*. The three terminal bases of the primer strand in the exonuclease active site from 3′–5′ are colored *green*, *cyan*, and *gray*, respectively. The two Mg^2+^ ions in the exonuclease active site are shown as *yellow spheres*. This figure shows that only three bases must melt for the primer to fully partition into the exonuclease active site, but this also requires rotation and translocation of the duplex DNA.

We prepared a starting model for MD simulation refinement from a crystal structure of T7 DNA polymerase bound to dsDNA in the polymerase active site. Missing residues were filled in, the system was solvated with TIP3P (3 site) waters (45), Mg^2+^ and Na^+^ ions were added at the concentrations used in kinetic experiments, then Cl^−^ ions were added to neutralize the system, and an initial energy minimization step was performed. A 500 ns unrestrained MD simulation showed that the structure was stable over the time course of the simulation (data not shown). We then tried copying the DNA substrate from the Klenow Fragment editing complex structure to the T7 DNA polymerase structure, but the simulations consistently failed due to high forces in the system, despite many optimization attempts. We therefore copied only the terminal 6 nucleotides of the primer from the Klenow Fragment structure into the T7 DNA polymerase structure and ran a 250 ns MD simulation to equilibrate energy minimizing the structure in the presence of ions and explicit water. Using the resulting coordinates, we were then able to copy the full dsDNA from the Klenow Fragment structure into the resulting T7 DNA polymerase structure and perform another MD simulation. At the end of the simulation, the dsDNA with ssDNA extending into the exonuclease active site was stable; however, catalytic Mg^2+^ ions had not equilibrated in the exonuclease active site during the simulation. Similar results were obtained with simulations on the polymerase active site, where ions did not equilibrate to the incoming dNTP during the simulation. Since ion binding may be slow relative to the timescale of the MD simulation, an alternate strategy was to use the coordinates of the ions in the homologous Klenow Fragment structure for starting ion positions for the T7 DNA polymerase simulation. Ion coordinates were copied from the Klenow Fragment structure (11), substituted with Mg^2+^, and another 250 ns simulation was performed. The two Mg^2+^ ions added to the exonuclease active site were stable during the simulation. The last frame of the trajectory was taken as the representative structure. Ultimately, we obtained the two desired structures, one with dsDNA in the polymerase active site (Fig. 4*A*) and another with a dsDNA bound with the primer strand projecting into the exonuclease active site (Fig. 4*B*). A linear interpolation morph between the two conformations is given in Video S1 as a first approximation to illustrate the path between the two states.

We emphasize that our structural model may have some limitations in terms of precise atomic interactions but nonetheless, the model provides reasonable constraints on how the DNA interacts with the enzyme in the exonuclease active site. As shown in Figure 4, *C* and *D*, three bases must melt from the DNA duplex for the primer to fully partition into the exonuclease active site, and this is consistent with estimates from homologous enzymes (42, 46). This preliminary pathway from the linear interpolation between the two active sites gives a structural model for the conformational dynamics underlying the movement of the DNA from the pol to the exo site. The process begins with a mismatch leading to stalling of processive polymerization. The DNA primer/template duplex is then twisted half a turn by rotation of the thumb domain, propagating the twisting motion through the thioredoxin-binding domain interactions with the downstream DNA, causing backtracking and unwinding of the replication fork, thereby aiding the transfer of the primer strand to the exonuclease active site. Surprisingly, only three base pairs melt, bringing the 3′ end of the primer from the polymerase to the exonuclease active site, a distance of 35 Å. Without backtracking, approximately five base pairs would need to be unwound to span the 35 Å distance.

### Phosphorothioate specificity of T7 DNA polymerase

**Figure 5:**
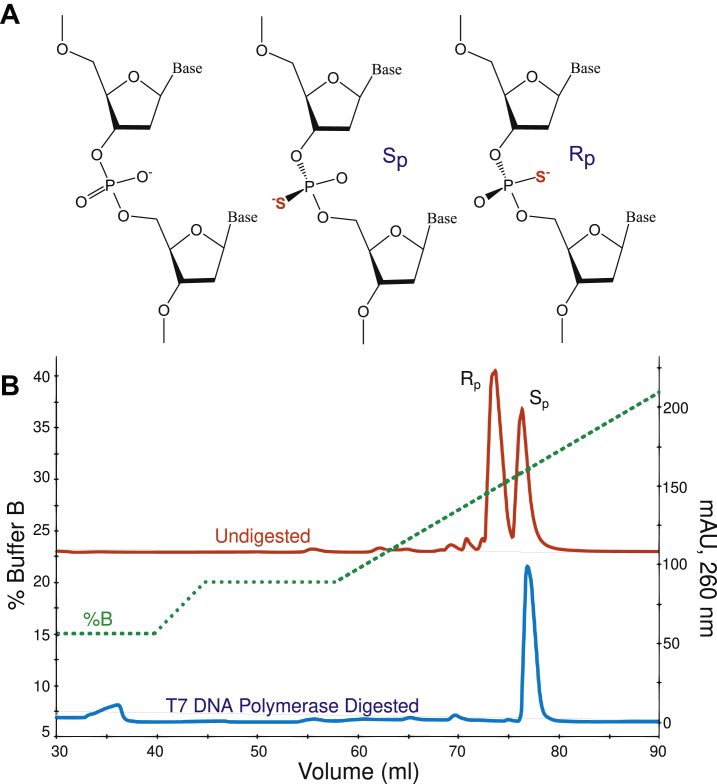
**Specificity of 3′–5′ exonuclease activity on phosphorothioate DNA**. *A*, diastereomers of phosphorothioate DNA. The two phosphorothioate substrates are shown on the *right*, with either an S_p_ or R_p_ configuration. The position of the sulfur atom is shown in *red*. *B*, specificity of T7 DNA polymerase exonuclease hydrolysis on phosphorothioate DNA. The isomers of a 6 nt oligonucleotide containing a phosphorothioate linkage at the 3′ phosphodiester linkage were resolved by reverse-phase high-performance liquid chromatography (HPLC) (*red chromatogram*). The elution gradient is given on the left-hand *y-axis* and the *dashed green line* gives the percentage of Buffer B in the separation (see Experimental procedures). Elution volume is given on the *x-axis* and milli-absorbance units at 260 nm are given on the right-hand *y-axis*. A sample of phosphorothioate oligonucleotide containing the racemic mixture of isomers was incubated with T7 DNA polymerase as described in the Experimental procedures and then resolved by reverse-phase HPLC (*blue chromatogram*) with the same conditions used for the untreated sample. During the incubation, the R_p_ isomer was completely hydrolyzed by the enzyme, while the S_p_ isomer remained.

Phosphorothioate containing oligonucleotides have been used to determine the stereospecificity of reactions and to stabilize nucleic acids used *in vivo* particularly in biotechnology applications (14). To test whether our active site homology modeling is valid for T7 DNA polymerase based on the Klenow Fragment structure, we determined the stereospecificity of the exonuclease hydrolysis reaction using an oligonucleotide containing a phosphorothioate linkage. For this experiment a 6 nt oligonucleotide (Table 1) with a phosphorothioate linkage at the 3′ terminal bond was used, with a racemic mixture of the two diastereomers (Fig. 5). First, the isomers of the racemic mixture were resolved by reverse-phase high-performance liquid chromatography (HPLC). As shown in Figure 5, near baseline separation of the isomers was achieved. Both major peaks were analyzed by MALDI-MS to confirm that they have the same m/z and are therefore isomers and not simply mixed products from the oligonucleotide synthesis (Figs. S2 and S3). It is well established in the literature that the S_p_ isomer elutes after the R_p_ isomer during reverse-phase separations on C18 columns, independent of the position of the phosphorothioate linkage (47, 48, 49); therefore, we assign the “fast” peak to the R_p_ isomer and the “slow” peak to the S_p_ isomer. The stereospecificity of the T7 DNA polymerase exonuclease proofreading domain was determined with the same 6 nt phosphorothioate racemic mixture. An aliquot of the oligonucleotide was incubated with T7 DNA polymerase to allow digestion of the single-stranded oligonucleotide by the 3′–5′ proofreading exonuclease. The digested sample was then resolved by reverse-phase HPLC, as above, to give the results shown in the blue trace in Figure 5. After digestion, only the S_p_ peak (“slow” peak) was present in the chromatogram indicating that the R_p_ isomer was completely hydrolyzed during the course of the reaction. Studies on other enzymes have shown that the S_p_ isomer blocks binding of metal ions to the exonuclease active site, significantly slowing the rate of hydrolysis (11). These results also indicate that the stereospecificity of the T7 DNA polymerase exonuclease hydrolysis reaction is the same as for all other known replicative DNA polymerases and is consistent with our homology/MD-based structural model for the T7 DNA polymerase exonuclease active site.

### Proposed catalytic mechanism for exonuclease hydrolysis by T7 DNA polymerase

**Figure 6:**
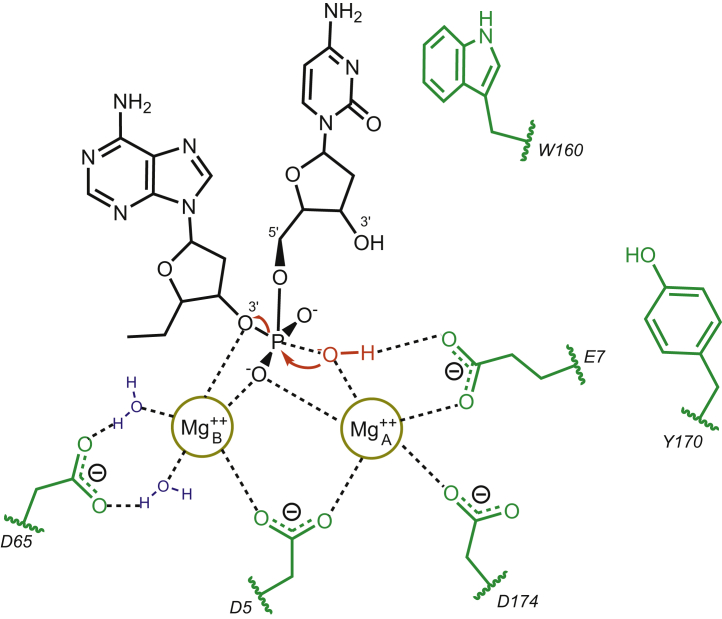
**Proposed exonuclease catalytic mechanism for T7 DNA polymerase.** The proposed catalytic mechanism for exonuclease hydrolysis by T7 DNA polymerase is shown, modified from (50). Amino acid side chains are shown in *green*, water molecules are shown in *blue*, the catalytic hydroxide ion is shown in *red*. The flow of electrons is shown by the *red arrows*.

Since the stereochemistry of the reaction is the same as for other polymerases that have been shown to have a conserved catalytic mechanism, based on previous work on Klenow Fragment of *E. coli* DNA polymerase I (50) we illustrate the catalytic mechanism for the exonuclease hydrolysis reaction for T7 DNA polymerase in Figure 6. In the exonuclease active site, W160 stacks with the 3′-terminal base of the primer strand. Other exonuclease active site structures have shown similar stacking of the 3′-terminal base with a hydrophobic residue, typically a phenylalanine residue (F473 in Klenow Fragment) (10, 11, 42, 51). Catalytic residues for T7 DNA polymerase *versus* other polymerases are given in Table S3. The implications of a tryptophan residue here are unclear, but it is apparent that some stacking of a hydrophobic protein residue with the terminal base helps properly align the terminal base for hydrolysis. In our proposed exonuclease mechanism, Mg^2+^ A interacts with a DNA backbone oxygen, D5, E7, D174, and the nucleophilic hydroxide ion. Mg^2+^ B coordinates a DNA backbone oxygen, D5, and D65 through a pair of water molecules. Previous studies suggest that Mg^2+^ A activates a water molecule, which acts as a nucleophile, attacking the phosphorous atom and forming a trigonal bipyramidal transition state, stabilized by Mg^2+^ B. Interestingly, our exo^−^ (D5A E7A) variant, used in previous studies to characterize kinetics at the polymerase active site, was designed based on gene sequence homology since no structural data were available at the time. Our proposed mechanism clearly demonstrates that the D5A E7A mutations will disrupt the metal-binding sites and lead to disorder in the exonuclease active site, explaining the large decrease (10^6^-fold) in exonuclease hydrolysis by this variant.

## Discussion

We have presented a kinetic analysis of the substrate specificity for the proofreading exonuclease function of T7 DNA polymerase and have proposed a feasible structural homology-based model to account for our results. We initially investigated the temperature dependence of the reaction, which was previously used to suggest that the substrate for the exonuclease reaction of *E. coli* DNA polymerase I is single-stranded DNA (7). We found that exonuclease activity at 4 °C was much slower than the reaction at 20 °C. This could be explained by increased fraying of single mismatched bases at the higher temperature as evidenced by the larger fraction of DNA binding from solution into the exonuclease active site at 20 °C relative to 4 °C, as well as faster transfer between the polymerase and exo sites as measured in the slower phase of the biphasic reaction kinetics (Fig. 1). However, our structural model (Fig. 4) suggests a significant, rate-limiting conformational change in the enzyme to facilitate the movement of the DNA from the polymerase to the exonuclease active sites. Since it is likely that the rate of the conformational change will also be temperature-dependent, one cannot explain the observed temperature dependence solely based on the thermodynamics of DNA melting. A small fraction of the DNA was resistant to hydrolysis at long times, as evidenced by a reduced amplitude of the slow reaction phase at lower temperature (Fig. 1). We propose that the reduced amplitude is due to the partitioning of fraction of the enzyme–DNA complex into an inhibited state. The identity of this state is unknown and may be more complex than the reaction given in the scheme in Figure 1, but adding this state is only needed to account for a small fraction of DNA that is resistant to cleavage to provide a complete minimal model sufficient to account for the kinetic data. Taken together, these data suggest that the enzyme assists in melting the mismatched duplex DNA, and it facilitates the underlying biophysical melting process arising from the mispair.

We previously demonstrated that the fidelity of polymerization does not significantly change as a function of temperature from 4 °C and 20 °C, although the rates of polymerization do increase slightly. Conversely, our data indicate that the contribution of the proofreading exonuclease to fidelity, at least for excision of terminal mispairs, is highly temperature-dependent. At 4 °C, the contribution of the exonuclease to overall fidelity appears to be negligible so the net fidelity is due mainly to reactions at the pol site (net fidelity ∼1 × 10^−6^). At higher temperatures, there is a greater contribution from the exonuclease proofreading reaction to fidelity so that at 20 °C, the exonuclease proofreading reaction contributes at least a factor of 1000 to fidelity and may contribute even more at 37 °C. A complete kinetic analysis of the correct incorporation, misincorporation, and exonuclease proofreading at various temperatures will be necessary to fully test this prediction. As described below, our data revealed multiple opportunities for proofreading due to increased rates of excision of buried mismatches compared with 3′-terminal mismatches, and this additional proofreading function contributes to fidelity, even at 4 °C.

### Thermodynamics

From the estimated rate constants for binding of DNA to the pol or exo sites (Tables 2 and 3 for 4 and 20 °C, respectively), we can calculate the net equilibrium constants for the transfer of the DNA from the pol to the exo site for DNA containing a single mismatch: *K*_*3*_ = 0.061 at 20 °C and 0.033 at 4 °C, indicating that 94 to 97% of the DNA will be bound to the pol site. In addition, from the estimated rate constants for transfer of DNA from the pol to the exo site, we can use the equilibrium constant to estimate the rate of transfer back from the exo to the pol site to be 48 s^−1^ and 8 s^−1^ at 20 and 4 °C, respectively. These rate constants for transfer of the DNA from the exo to the pol site allow ample opportunity for excision of the terminal base pair from the primer strand, which occurs at ∼1000 s^−1^ (4). The equilibrium constant for partitioning into the exo site for DNA without a mismatch is only 0.0087 at 20 °C, so a single mismatch increases the equilibrium constant sevenfold. With four terminal mismatches, the equilibrium constant is 1, so the increased fraying of the duplex DNA due to multiple 3′-terminal mismatches favors binding to the exo site. The net free energy difference from transfer of the DNA from the pol to exo site is a function of multiple events, including DNA unwinding.

Although we only have data at two temperatures, we can use these results to provide a crude estimate of the thermodynamic parameters governing the transfer of DNA from the pol to the exo site: ΔG^o^ = 6.8 kJ/mol (at 20 °C) and ΔH^o^ = 29 kJ/mol. Thus, the reaction is unfavorable in the enthalpy change but favorable in the entropy change for the reaction. These preliminary estimates require further investigation, but they suggest that transfer of DNA from the pol to the exo site requires the input of heat, potentially from the breaking of base pairs and stacking interactions in the DNA. They also suggest that the binding of DNA to the exo site is less ordered with more degrees of freedom than the complex with duplex DNA. However, interpretation of these results requires consideration of the significant role of waters of hydration in the two states in addition to the thermodynamics of melting the duplex, binding of the primer stand at the exo site, and extensive structural changes in the enzyme. Simple conclusions in the absence of more rigorous analysis are not valid. Nonetheless, the data support the conclusion that binding of duplex DNA at the pol site is thermodynamically and kinetically favored so that 95% of the time the DNA with a single mismatch remains at the pol site and makes occasional excursions to the exo site. Without a mismatch, the DNA remains at the pol site 99% of the time. Selectivity of the exonuclease to remove mismatches is largely a function of stalling of the polymerization reaction to allow time for the DNA to flip into the exo site (4).

### Multiple opportunities for proofreading

In the initial experiments, we measured exonuclease activity on DNA substrates containing various numbers of terminal mismatches, as reported previously to characterize the exonuclease activities of other polymerases (4, 38). Analysis of exonuclease hydrolysis of DNA containing multiple mismatches is a useful approach to probe the biophysics of proofreading even though it does not represent a physiologically significant state because of the low probability of forming multiple sequential mismatches. Rather, it has been shown that once a mismatch is incorporated at the polymerase active site, incorporation of a second mismatched nucleotide on top of the mismatch is unlikely, and any extension will be with correct base pairs, not an additional mismatch (17). To monitor a more physiologically relevant reaction, we investigated the kinetics of exonuclease hydrolysis on DNA substrates containing mismatches buried by correct bases. We found that mismatches buried at the n-1 through n-3 position (buried by 1–3 correct base pairs) were removed much more efficiently than 3′-terminal mismatches. This indicates that even if the mismatch was extended by several bases, the kinetic partitioning still favors excision rather than extension. To test whether this phenomenon afforded significant additional opportunities for proofreading, we measured the rates of extension of various buried mismatches to allow comparison with the rates of excision. We found that even at high nucleotide concentrations (1 mM) and relatively long incubation times (up to 3 min), the enzyme would not extend mismatches that were buried at the n-1 or n-2 position (Fig. 3). We did observe extension for the n-3 buried mismatch, albeit at a slow rate (13.2 s^−1^). These results further underscore the interplay between the proofreading exonuclease domain and the polymerase active site and demonstrate that there are multiple opportunities for proofreading before a mismatch is fully buried to afford stable incorporation into the genome. The net fidelity contribution of the exonuclease is therefore a function of the product of probabilities of excision *versus* extension for up to at least three base pairs on top of a mismatch. Using the rates determined here, we add that beyond the 200-fold increase in fidelity from excising terminal mismatches incorporated at the polymerase active site, the high rate of excision of buried mismatches *versus* the extremely slow rates of extension add an additional factor of at least 50 to yield a net contribution of 10^4^ for proofreading toward the net fidelity for this enzyme. Although local sequence context effects may significantly decrease the overall contribution of the proofreading exonuclease to fidelity, our results demonstrate a large effect of the exonuclease in improving the fidelity of DNA replication so even reduced proofreading efficiency in certain sequence contexts may still constitute a major contribution to fidelity.

### Structure

To provide a structural framework for understanding the exonuclease function for T7 DNA polymerase, we built a model based on the homologous Klenow Fragment structure (42) and then used all atom MD simulations to refine the structure. Despite a sequence identity of generally less than 15% for related exonuclease domains (9), they all exhibit high structural similarity and share a set of well-conserved carboxylates at the exonuclease active site that function as ligands for the two divalent metal ions required for catalysis. Despite this high level of conservation, the exonuclease activity varies greatly among the well-characterized enzymes. The exonuclease activities of T7 and T4 DNA polymerases are at least 100 times greater than that for Klenow fragment (3). In our homology model based on the structure of Klenow, we also observe the carboxylates that coordinate two metal ions in the exonuclease active site, but there are some key differences. In the structure of Klenow Fragment, Y497 points toward the phosphorous and was proposed to contribute to the hydrolysis reaction (10, 11, 42, 51). In T4 polymerase, the corresponding residue is tyrosine (Y320), which points away from the active site (9) and does not appear to directly contribute to the reaction. Also, different residues form the binding pocket for the 3′ terminal base of the substrate, and the 3′ terminal base is rotated approximately 60 degrees relative to the position of the corresponding base in Klenow Fragment. In our structural model, tyrosine (Y170) also points away from the exonuclease active site, like the T4 DNA polymerase structure. In both the T4 and Klenow Fragment structures, a phenylalanine residue (F120 in T4 pol, F473 in KF) stacks with the terminal base and positions it for hydrolysis. In the T7 DNA polymerase model, W160 stacks with the terminal base. The implications of this change from Phe to Trp are still unknown and warrant further investigation, but the role of a hydrophobic residue in positioning the 3′-terminal base of the primer strand appears to be conserved.

Our model reveals significant structural changes in the enzyme when the DNA primer strand transfers from the pol site into the exonuclease active site. Similar to a recent study on *E. coli* DNA polymerase III that used targeted MD simulations to generate the minimum energy path between the exonuclease and polymerase complexes (46), our model suggests that the DNA backtracks. Rotation of the thumb domain and the thioredoxin-binding domain that interacts with downstream DNA serves to unwind the DNA. The template strand is held relatively fixed by residues in the fingers domain, allowing the primer terminus to unzip and partition into the exonuclease active site when the downstream DNA rotates. We have created a linear interpolation between the two structures (Video S1) that illustrates this large conformational change in the enzyme–DNA complex, but more sophisticated methods could be used in the future to generate a minimum energy path.

In summary, we have characterized the substrate specificity for T7 DNA polymerase exonuclease activity and provided a minimal kinetic model that accounts for the observed reactions. These techniques should be applicable to more complex DNA replication enzymes with proofreading exonuclease functionality, and future studies could further probe the reaction pathway with more sophisticated techniques such as fluorescence to monitor the partitioning of the primer strand. Solving a crystal structure with DNA in the exonuclease active site or more sophisticated computational methods could provide more fine atomic details about the exonuclease hydrolysis reaction for this enzyme.

## Experimental procedures

### Enzymes, oligonucleotides, and reagents

Wild-type (exo^+^) T7 DNA polymerase, exo- (D5A E7A) T7 DNA polymerase, and thioredoxin were expressed in *E. coli* and purified as previously described (15) with one modification: the D5A and E7A mutations (21) in pcI^ts^-(T7 GP5x^−^, trxA) were back mutated to the wild-type D5 and E7 with site-directed mutagenesis to make the expression plasmid for the wild-type exo^+^ enzyme, pcI^ts^-(T7 GP5x^+^, trxA). All experiments in this paper were performed with the wild-type (exo^+^) T7 DNA polymerase unless otherwise indicated. All DNA oligonucleotides were synthesized by Integrated DNA Technologies with standard desalting. Phosphorothioate oligos were purchased as a racemic mixture of the two diastereomers. Oligos used in kinetics assays were further purified in house by denaturing PAGE to >98% purity as determined by capillary electrophoresis. The prGrG (RNA) internal standard was synthesized by Dharmacon. Purified oligonucleotide stocks were stored in 66.2 Buffer (6 mM Tris-HCl pH 7.5, 6 mM NaCl, 0.2 mM EDTA) at −20 °C. Double-stranded DNA substrates were prepared by mixing the primer and template strands at a 1:1 M ratio in Annealing Buffer (10 mM Tris-HCl pH 7.5, 50 mM NaCl, 1 mM EDTA), heating to 95 °C for 3 min, then cooling slowly to room temperature over the course of 1 h. Oligos used as internal standards for MALDI were used without further purification. BSA was purchased from New England Bio Labs. Diammonium hydrogen citrate and 3-hydroxypicolinic acid were purchased from Acros Organics. Triethylammonium bicarbonate pH 8.5 (TEAB) buffer was purchased from Sigma Aldrich. Acetonitrile, acrylamide, urea, and other buffer components were purchased from Thermo Fisher Scientific.

### Phosphorothioate diastereomer separation by HPLC

Buffer A (50 mM TEAB pH 8.5) and Buffer B (50 mM TEAB pH 8.5, 30% [v/v] acetonitrile) were prepared fresh before HPLC separations. First, a sample of undigested 6mer-PThio (∼50 μg) was diluted into 10% Buffer B and injected onto a Vydac 218TP54 C18 column, equilibrated in 10% Buffer B. Absorbance at 260 nm was monitored on an AKTA Pure HPLC, and the run was performed at a constant pressure of 5 MPa at room temperature (∼25 °C). After injection, the column was washed with 10 ml of 15% Buffer B, sufficient to bring the absorbance to baseline. Next a gradient to 20% Buffer B was performed over 5 ml, followed by washing with 10 ml of 20% Buffer B. Finally, an elution gradient from 20 to 40% Buffer B over 35 ml was performed to elute the bound oligonucleotides. Near baseline separation of the two isomers was achieved (Fig. 5), and the identity of each peak was confirmed by MALDI-MS (see below).

To test the stereospecificity of the T7 DNA polymerase exonuclease domain, a 1 ml reaction containing 0.5 μM T7 DNA polymerase, 29 μM 6mer-PThio, and 12.5 mM Mg^2+^ in T7 Reaction Buffer (40 mM Tris-HCl pH 7.5, 1 mM EDTA, 50 mM NaCl, 1 mM DTT) (1, 15) was incubated for 30 min at 20 °C in a circulating water bath. The reaction was quenched by adding EDTA to 0.2 M. Protein was denatured by incubating the quenched reaction at 80 °C for 10 min, and then denatured protein was pelleted by centrifugation at maximum speed in a tabletop microcentrifuge for 10 min. Before HPLC, the sample was cleaned up with a SepPak C18 cartridge (Waters). The cartridge was first flushed with 10 ml of 100% acetonitrile and then flushed with 10 ml of 10% Buffer B. The sample was then applied to the column at approximately 1 ml/min, and the column was washed with 10 ml of 10% Buffer B. Bound oligonucleotide was then eluted with approximately 5 ml of 50% Buffer B. The sample was then diluted fivefold with Buffer A before loading on the HPLC/C18 column and performing the separation using the same conditions as above.

### Mass spec

A solution of 100 mg/ml diammonium hydrogen citrate was prepared fresh in dH_2_O. A stock of 50 mg/ml 3-hydroxypicolinic acid was prepared fresh in 50% (v/v) acetonitrile before use. Solutions of 3-hydroxypicolinic acid and ammonium citrate dibasic were mixed at a 10:1 ratio before use. Samples contained 10 μM oligonucleotide of interest and 5 μM each internal standard oligonucleotide listed in Table S2. The internal standard oligos range from 2 nt through 10 nt in length for a total of four points for internal calibration. An aliquot of 0.6 μl of the matrix solution was spotted on wells on a steel plate and dried to completion at room temperature. Once dry, 0.6 μl of sample was spotted on top of the dried matrix and dried to completion at room temperature. Before sample data collection, the instrument was calibrated with a calibration mix spotted near the sample spots on the plate. Positive ion mode was used with the reflector detector. Sample data collection was performed using the same instrument settings used for initial calibration. Data were exported for further analysis with the program mMass (52). In mMass, peaks were identified using a signal/noise threshold of 10, relative intensity threshold of 15%, and a picking height of 100. Identified peaks not corresponding to the monoisotopic peak were deleted, and no baseline correction or smoothing was applied. The peaks were then searched against a database of the internal standard and sample oligonucleotide, and all major peaks were successfully identified within 50 ppm error. Internal calibration using a quadratic function was then performed using the matched compounds to further reduce errors to less than 20 ppm.

### Kinetics experiments

All experiments were performed in T7 Reaction Buffer (40 mM Tris-HCl, pH 7.5, 50 mM NaCl, 1 mM EDTA, 1 mM DTT) (15). In some experiments, 12.5 mM Mg^2+^ was present in both mixtures before starting the reaction, and for other experiments, Mg^2+^ was present in only one syringe at 2× concentration to prevent degradation of the DNA by the highly active exonuclease domain during the preincubation stage. In either case, the final Mg^2+^ concentration during the reaction was 12.5 mM. Rapid quench experiments were performed on an RQF-3 instrument (KinTek), equipped with a circulating water bath for temperature control. Reaction Buffer was loaded into the drive syringes, and 0.6 M EDTA was loaded into the quench syringe. For samples separated by PAGE, Formamide Loading Buffer (5 % [w/v] sucrose, 90% [v/v] formamide, 0.025% [w/v] bromophenol blue, 0.025% [w/v] xylene cyanol, 10 mM EDTA) was added to each sample at a 1:2 ratio of Formamide Loading Buffer to sample, followed by denaturation at 98 °C for 2 min and separation on preheated 15% polyacrylamide sequencing gels containing 7 M urea for approximately 3 h at 50 °C. Gels were scanned on a Typhoon FLA 9500 laser scanner (GE Healthcare) with the FAM fluorescence filter, and the resulting images were quantified with Image Quant software (GE Healthcare). Sample separation by capillary electrophoresis was performed as previously described (20). Concentrations of reaction components given in the text are final concentrations after mixing unless otherwise noted.

### Data fitting and analysis/figure preparation

Kinetic data were fit by simulation in KinTek Explorer version 10 (53, 54). Models used for fitting are given in Figures 1 and 2, and locked rate constants are described in the main text and in the footnotes of Table 2, Table 3, Table 4, Table 5. Chemical structures were prepared with ChemDraw Professional 16.0 (PerkinElmer Informatics). Inkscape was used to prepare figures. Pymol was used to make structure figures. Graphs of processed data were created in Microsoft Excel. Kinetic figures were prepared with KinTek Explorer. Calculations of Flux in comparing fraction of reactions proceeding *via* alternative branched pathways were performed using a dynamic partial derivative analysis during data fitting based on numerical integration of the rate equations using KinTek Explorer software (53, 54).

### Flux calculations

To quantify the relative partitioning of intermediates between branched pathways, we used the *Flux Calculation* feature in KinTek Explorer whereby the partitioning was derived numerically from the partial derivatives for the competing reactions. For example, for the relative partitioning of DNA between binding to the exo and pol site (Fig. 1), we calculate the percentage of DNA binding to the exo site *versus* the pol site (Table 2) using the syntax, *D::ED*_*x*_ for the rate of DNA binding to the exo site and *D::ED*_*p*_ for the rate of binding to the pol site. The percentage DNA binding to the exo site is then calculated as 100∗(*D::ED*_*x*_/(*D::ED*_*x*_ + *D::ED*_*p*_)). The percentage of DNA bound at the pol site (*ED*_*p*_) partitioning to *ED*_*x*_ *versus* to *EI* was calculated as 100∗(ED_p_::ED_x_/(ED_p_::ED_x_ + ED_p_::EI)).

### Homology modeling/molecular dynamics simulations

Before homology modeling, an initial structure for the binary T7 DNA polymerase enzyme–DNA (*ED*_*p*_) complex was prepared from pdb: 2ajq. There are two copies of the binary *ED*_*p*_ complex in the structure, and the copy with more resolved DNA was used. In the crystal structure, residues 300 to 314 in the thioredoxin-binding domain were not resolved so they were modeled in with the program Coot (55). The sequence of the DNA was changed in Pymol to match the sequence of the DNA substrate used in kinetics experiments and extended to give 27 bases of primer and 45 bases of template DNA, using Chimera (56) to generate B form DNA for the duplex DNA extension and a random coil for the template strand extension. After these corrections, remaining steps for system preparation were performed with GROMACS v 5.0.4 (44). Bonded and nonbonded interactions between atoms were parameterized with the AMBER ff99SB force field (57). The protein was placed in a triclinic box 1.2 nm from the protein surface, and TIP3P (45) water molecules were added. Na^+^ and Mg^2+^ were added based on the box volume, and concentration of these ions used in experiments. Cl^−^ ions were then added to neutralize the system. To remove the bad contacts that may arise due to random placement of ions and water, energy minimization was performed with the steepest descent algorithm for 1000 steps.

### Preparation of starting dsDNA in polymerase active site structure

To utilize MD simulations and to generate a stable starting binary *ED*_*p*_ complex structure (with DNA in the polymerase active site), the system was initially equilibrated in the NPT ensemble at 293 K, 1 bar, with position restraints on all atoms except water and ions. We used a time step of 1 fs for integrating the equations of motion. Particle Mesh Ewald sum (58) was used to calculate long-range electrostatics. Temperature coupling with a modified Berendsen thermostat was used with a time constant of 2 ps and a reference pressure of 1 bar. Periodic boundary conditions were used in the x, y, and z planes. The covalent bond lengths of the water and the enzyme were constrained by SETTLE (59) and LINCS (60) algorithms, respectively, on all bonds. Neighbor searching was done with a grid updating the list every 10 fs with a 10-Å short-range neighbor cutoff. Short-range electrostatics were cut off at 10 Å, and short-range van Der Waals cutoff was also set to 10 Å. During the simulation, coordinates were saved every 20 ps. Initial equilibration in the NPT ensemble was performed for a total of 2 ns. Next, equilibration of ions in the NVT ensemble was performed for 50 ns with a time step of 1 fs with the leapfrog integrator. Position restraints on all atoms, except ions, water, and the single-stranded template DNA strand extension were used in this step. Electrostatics were calculated using the Verlet cutoff scheme with PME for long-range electrostatics with the same time constant used previously. Pressure coupling was turned off during this simulation. Finally, a production run in the NVT ensemble was performed by removing all position restraints, increasing the time step to 2 fs, and running the simulation for a total of 500 ns. Tools in GROMACS were used to correct periodic boundary conditions at the end of the production run for visualization. The last frame of the simulation is shown in Figure 4*A*.

### Preparation of ssDNA in exonuclease active site intermediate structure

Klenow Fragment structure pdb: 1kln (22), containing Klenow Fragment bound to dsDNA with the primer strand in the exonuclease active site, was used as it was the closest structure available in the protein data bank for homology modeling. The structure was overlayed with the binary T7 DNA polymerase structure with DNA in the polymerase active site created above, and the terminal 6 bases of the primer strand in the Klenow Fragment structure were extracted, modified to match the sequence used in kinetics experiments, and copied to the T7 DNA polymerase binary complex structure with the double-stranded DNA removed. The resulting structure went through the system preparation steps above including solvation, ion addition, and energy minimization with tools available in GROMACS and the same force field. After system preparation, equilibration steps in the NPT and NVT ensembles were performed as above, followed by a 250 ns NVT production simulation run. The final frame was corrected as described above and used in the next step.

### Preparation of dsDNA in exonuclease active site structure

Next, the T7 DNA polymerase structure with dsDNA in the exonuclease active site structure was prepared using the T7 DNA polymerase/ssDNA in exonuclease active site structure obtained above. The dsDNA from the Klenow Fragment structure (pdb: 1kln (22)) was again used, but this time the entire dsDNA substrate was copied to the T7 DNA polymerase/ssDNA in exonuclease active site structure without the 6 nt ssDNA. The dsDNA was extended using the structure with dsDNA in the polymerase active site to give 27 bases of primer and 31 bases of template DNA. The system was prepared as above by solvation, ion addition, and energy minimization in GROMACS with the same force field. Ions were equilibrated as above, and the production simulation was ran for 250 ns. In this time frame, Mg^2+^ ions had not equilibrated into the exonuclease active site. A second simulation was therefore performed using the first dsDNA in the exonuclease active site structure as a starting point. The positions of ions (1× Zn^2+^ and 1× Mg^2+^) from pdb: 1kfs (45) were used to approximate the Mg^2+^ ion positions for T7 DNA polymerase exonuclease active site and copied to the T7 DNA polymerase structure. The system was prepared for MD simulation with the same steps described above, then a 250 ns production simulation was performed. The ions in the exonuclease active site remained bound during the entire simulation. The last frame of the trajectory was selected and is shown in Figure 4.

## Data availability

The exonuclease proofreading complex of T7 DNA polymerase has been deposited to modelarchive.org and can be accessed at https://modelarchive.org/doi/10.5452/ma-1e6bb.

## Supporting information

This article contains supporting information (17).

## Conflict of interest

K. A. J. is president of KinTek Corporation, which provided the RQF-3 rapid quench flow instrument and KinTek Explorer software used in this study.
