# Supplementary material for: Substrate specificity and proposed structure of the proofreading complex of T7 DNA polymerase
Source: J Biol Chem. 2022 Jan 22;298(3):101627. doi: 10.1016/j.jbc.2022.101627 (PMC8867116; doi:10.1016/j.jbc.2022.101627)
Supplement: Supplemental Figures S1–S3 and Tables S1–S3 [file mmc1.pdf]

<u>Substrate specificity and proposed structure of the proofreading complex of T7 DNA polymerase</u>

Tyler L. Dangerfield, Serdal Kirmizialtin, Kenneth A. Johnson

Supplemental Information contents:

Table S1: Mismatch Extension Kinetic Parameters
Table S2: Mass spec internal standard oligonucleotides
Table S3: Exonuclease Active Site Residues in Klenow Fragment, T7 DNA Polymerase, and T4 DNA Polymerase
Figure S1: Mismatch extension measurements figures
Figure S2: Mass spectrum from MALDI-MS on the “fast” phosphorothioate peak.
Figure S3: Mass spectrum from MALDI-MS on the “slow” phosphorothioate peak
Video S1: Morph of T7 DNA polymerase structures with DNA in the exonuclease active site and DNA in the polymerase active site.

Table S1: Mismatch Extension Kinetic Parameters

**dNTP**	dCTP*	dTTP	dTTP
**Templating Mismatch X:Y (X=primer, Y=template)**	A:A	T:T	T:G
**k_{cat} (s^{-1}) [lower,upper]**	0.025	0.19 [0.18, 0.20]	1.8 [1.3, 3]
**K_m (μM) [lower,upper]**	87	415 [380, 450]	5,000 [3,500, 8600]
**k_{cat}/K_m (M^{-1} s^{-1}) [lower,upper]**	290	460 [400, 530]	360 [150, 860]

* values from (1)

Table S2: Mass spec internal standard oligonucleotides

Oligo Name	Sequence 5’→3’	Extinction Coefficient, 260 nm (M^{-1} cm^{-1})
2mer	prGrG	21,600
5mer	TCAAC	47,800
7mer	ACTCAAC	68,100
10mer	CCAACTCAAC	93,100

rN = ribonucleotide, p = 5’ phosphorylated

Table S3: Exonuclease Active Site Residues in Klenow Fragment, T7 DNA Polymerase, and T4 DNA Polymerase

Klenow Fragment Residue	T4 DNA Polymerase Residue	T7 DNA Polymerase Residue
D424	D219	D65
D355	D112	D5
D501	D324	D174
E357	E114	E7
Y497	Y320	Y170
F473	F120	W160

$$ED + dTTP \underset{k_{-1}}{\overset{k_1}{\rightleftarrows}} EDT \xrightarrow{k_2} EP + PP_i$$

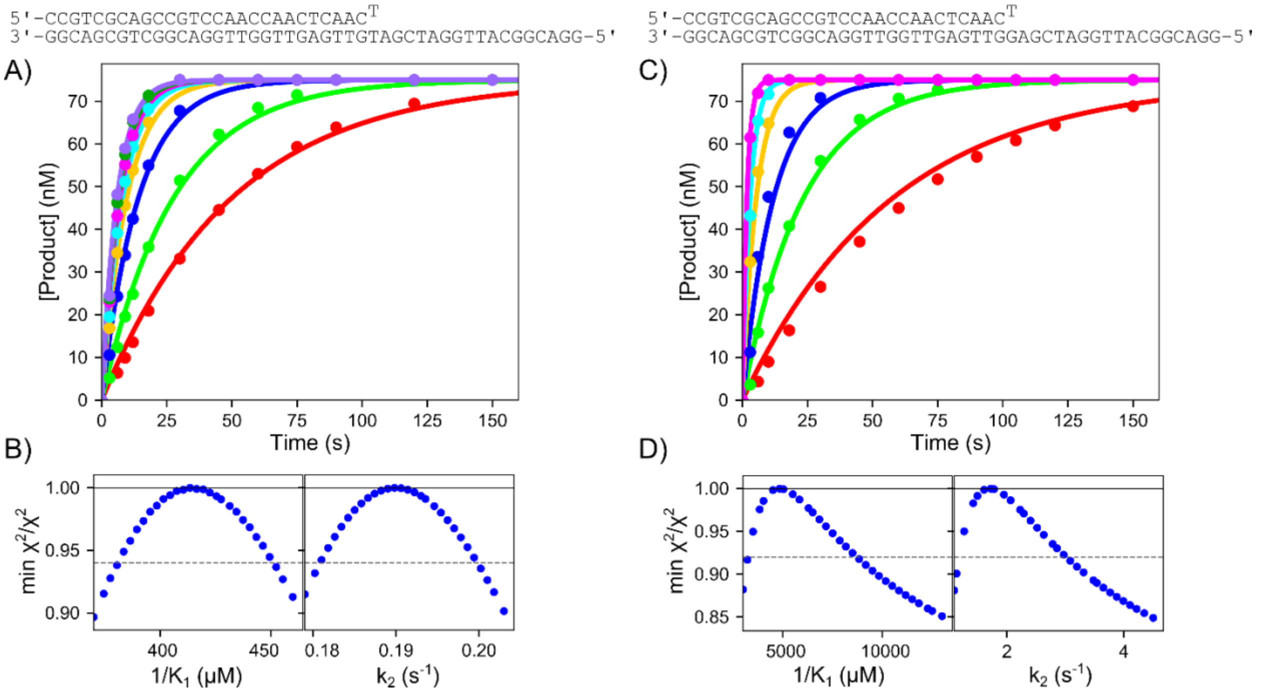

<u>Figure S1: Mismatch extension measurements</u>. The model used for data fitting is shown at the top of the figure. In the fitting, k_1 was locked at 10 µM^{-1} s^{-1} and k_{-1} and k_2 were allowed to float. Synthetic oligonucleotide substrates used for each mismatch extension reaction are shown above the corresponding concentration versus time plot. <u>A) Time course of T:T mismatch extension with dTTP.</u> A solution of exonuclease deficient 225 nM T7 DNA polymerase E514Cou, 4.5 µM thioredoxin, 0.1 mg/ml BSA, and 75 nM FAM-DNA was mixed with 0.05 – 3 mM Mg^{2+}-dTTP and 12.5 mM Mg^{2+} to start the reaction. Time points were quenched with 0.3 M EDTA. <u>B) Confidence contours for kinetic parameters for T:T mismatch extension with dTTP.</u> Confidence contours for 1/K_1 and k_2 are shown, with the dashed line corresponding to the χ^2 threshold corresponding to the 95% confidence interval. The resulting parameters are given in Table S1. <u>C) Time course of T:G mismatch extension with dTTP.</u> A solution of exonuclease deficient 225 nM T7 DNA polymerase E514Cou, 4.5 µM thioredoxin, 0.1 mg/ml BSA, and 75 nM FAM-DNA was mixed with 0.05 – 2.5 mM Mg^{2+}-dTTP and 12.5 mM Mg^{2+} to start the reaction. Time points were quenched with 0.3 M EDTA. <u>D) Confidence contours for kinetic parameters for T:G mismatch extension with dTTP.</u> Confidence contours for 1/K_1 and k_2 are shown, with the dashed line corresponding to the χ^2 threshold corresponding to the 95% confidence interval. The resulting parameters are given in Table S1.

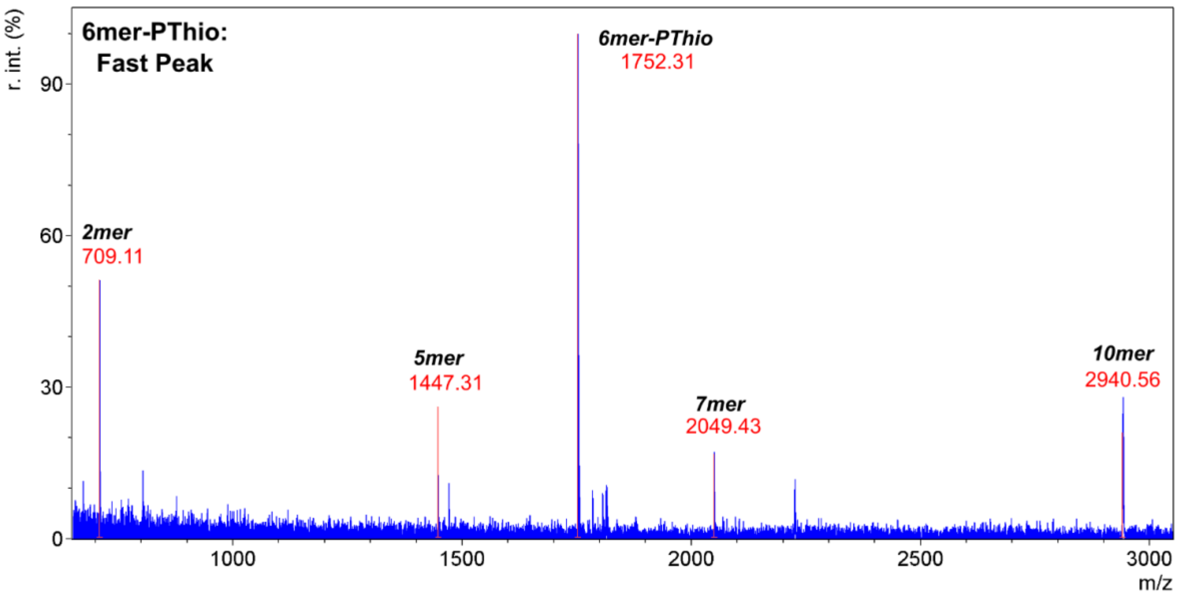

<u>Figure S2: Mass spectrum from MALDI-MS on the "fast" phosphorothioate peak.</u> Samples contained 10 µM oligo of interest and 5 µM internal standard oligos of 2,5,7, and 10 nt in length (see Table S1). Samples were applied on top of a layer of dried matrix (3-hydroxypicolinic acid/ diammonium hydrogen citrate) and were analyzed in positive ion mode. Raw data are shown in blue and identified peaks are shown as red lines with their respective m/z above the peak. After identifying the major peaks in the spectrum, internal calibration was used to bring the error to less than 20 ppm.

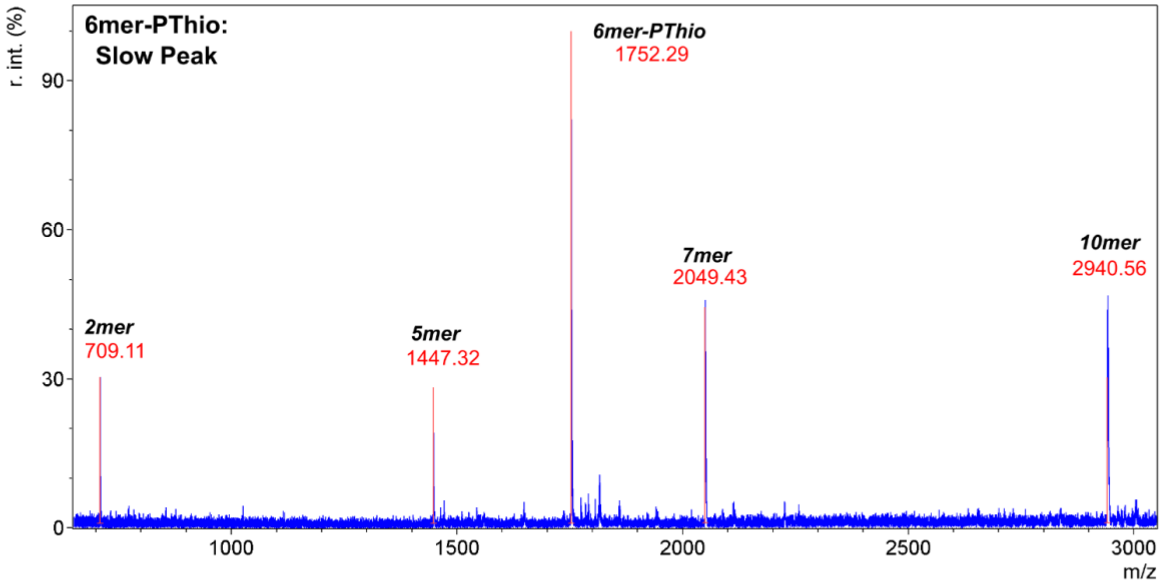

<u>Figure S3: Mass spectrum from MALDI-MS on the "slow" phosphorothioate peak.</u> Samples contained 10 µM oligo of interest and 5 µM internal standard oligos of 2,5,7, and 10 nt in length (see Table S1). Samples were applied on top of a layer of dried matrix (3-hydroxypicolinic acid/ diammonium hydrogen citrate) and were analyzed in positive ion mode. After identifying the major peaks in the spectrum, internal calibration was used to bring the error to less than 20 ppm.

Video S1: Morph of T7 DNA polymerase structures with DNA in the exonuclease active site and DNA in the polymerase active site. Bases in the primer strand are colored purple and bases in the template strand are colored yellow. Thioredoxin is colored orange. Domains in T7 gene product 5 are colored as follows: thioredoxin binding domain, grey; thumb domain, green; fingers domain, blue; palm domain, red; exonuclease domain, cyan.

References

1. Wong, I., Patel, S. S., and Johnson, K. A. (1991) An induced-fit kinetic mechanism for DNA replication fidelity: direct measurement by single-turnover kinetics. *Biochemistry* **30**, 526-537
